# Characterization of Photosynthetic Phenotypes and Chloroplast Ultrastructural Changes of Soybean (*Glycine max*) in Response to Elevated Air Temperatures

**DOI:** 10.3389/fpls.2020.00153

**Published:** 2020-03-06

**Authors:** Matthew T. Herritt, Felix B. Fritschi

**Affiliations:** ^1^US Arid Land Agricultural Research Center, United States Department of Agriculture, Agricultural Research Service, Maricopa, AZ, United States; ^2^Division of Plant Science, University of Missouri, Columbia, MO, United States

**Keywords:** chlorophyll fluorescence, gas exchange, transmission electron microscopy, heat stress, soybean

## Abstract

Heat stress negatively affects photosynthesis in crop plants. Chlorophyll fluorescence provides information about the efficiency of the light-dependent reactions of photosynthesis and can be measured non-destructively and rapidly. Four soybean (*Glycine max*) genotypes were grown in controlled environments at 28/20°C (control), followed by imposition of control, 38/28°C, and 45/28°C day/night temperature regimes for 7 days. Coordinated chlorophyll fluorescence, gas exchange, and chloroplast ultrastructure measurements were conducted over the course of the 7-day temperature treatments and revealed contrasting responses among the different genotypes. Although generally similar, the extent of the impact of elevated temperatures on net photosynthesis differed among genotypes. Despite dramatic effects on photosynthetic light reactions, net photosynthetic rates were not reduced by exposure to 45°C on the 1^st^ day of treatment imposition. Temporal dynamics of light reaction characteristics over the course of the 7-day heat-wave simulation revealed distinct responses among the genotypes. Similarly, chloroplast ultrastructure examination identified contrasting responses of DT97-4290 and PI603166, particularly with respect to starch characteristics. These changes were positively associated with differences in the percent area of chloroplasts that were occupied by starch grains. Elevated temperature increased number and size of starch grains on the 1^st^ day of DT97-4290 which was coordinated with increased minimum chlorophyll fluorescence (F_0_) and reduced leaf net CO_2_ assimilation (A). Whereas on the 7^th^ day the elevated temperature treatment showed reduced numbers and sizes of starch grains in chloroplasts and was coordinated with similar levels of F_0_ and A to the control treatment. Unlike starch dynamics of PI603166 which elevated temperature had little effect on. The genotypic differences in photosynthetic and chloroplast ultrastructure responses to elevated temperatures identified here are of interest for the development of more tolerant soybean cultivars and to facilitate the dissection of molecular mechanisms underpinning heat stress tolerance of soybean photosynthesis.

## Introduction

Plants have the ability to cool their leaves below that of the air temperature. In areas like the US Southwest, leaf temperatures of plants can be up to 10°C cooler than air temperatures (Idso et al., 1982). However, in conditions where leaf cooling cannot keep pace with increasing air temperatures, elevated leaf temperatures can lead to reductions in photosynthesis (Fischer et al., 1998). Reduction of net photosynthesis with increased temperatures have been observed for numerous species, including tomato (*Lycopersicon esculentum*) (Abdelmageed and Gruda, 2009), cotton (*Gossypium barbadense* L.) (Carmo-Silva et al., 2012), wheat (*Triticum aestivum* L.) (Hassan, 2006), and soybean [*Glycine max* (L.) Merr.] (Vu et al., 1997). The impacts of elevated temperature on photosynthesis have primarily been attributed to inactivation of Rubisco mediated by effects on Rubisco activase stability (Crafts-Brandner and Salvucci, 2000; Sharkey et al., 2001; Salvucci and Crafts-Brandner, 2004; Kurek et al., 2007) and photosynthetic electron transport (Yamane et al., 1997; Zhang et al., 2010).

The light-dependent reactions of photosynthesis allow plants to harvest and convert light energy into chemical energy, including ATP and NADPH for use in carbon fixation reactions. Central to the light-dependent reactions are photosystems I and II (PS1 and PS2). The photosystems are the central hubs where the energy from absorbed light is used to excite electrons. Electrons originate from water and are transported from PS2 *via* mobile electron carriers and cytochrome b6f to PS1. The photosystems, cytochrome b6f and the mobile electron carriers as well as the bulk of the accessory proteins involved with the light-dependent reactions are located either within or near the thylakoid membrane (Maxwell and Johnson, 2000; Friso et al., 2004).

The location of the light-dependent reactions in and around the thylakoid membrane is critical for photosynthesis. The thylakoid membrane serves several roles for the light-dependent reactions, including maintenance of the proton gradient that is used for ATP production (Alberts et al., 2002). The interaction between the thylakoid membrane and the photosystems is also crucial for the light dependent reactions of photosynthesis (Loll et al., 2007). To balance the production of ATP and NADPH, the two photosystems are spatially separated in the thylakoid membrane (Albertsson, 2001). The separation of the photosystems leads to stacking of the thylakoid membranes rich in PS2 through electrochemical and protein-protein interactions (Andersson et al., 2003; Chow et al., 2005). The stacked thylakoid, grana, allow for regions that are concentrated with PS2 complexes and some PS1 complexes. The rest of the thylakoid that does not form into grana contains many more PS1and ATP synthases than grana. This separation of complexes and the organization of the thylakoid membrane allows for efficient NADPH production within the grana and ATP production primarily in the non-appressed regions (Kirchhoff et al., 2013). Grana stacking is often associated with efficient light-dependent reactions (Jenny et al., 2003). When grana stacking is interfered with under heat stress, the efficiency of the light reactions is reduced (Xu et al., 2006). Damage to the D1 protein of PS2 reduces the efficiency of NADPH production and this damage can disrupt the forces responsible for stacking (Fristedt et al., 2009). The full OJIP traits from the experiment can be found in **Supplementary Table 1**.

The critical role of chloroplast ultrastructure for the light-dependent reactions, warrants detailed investigations to assess changes that may occur because of heat stress. Indeed, previous investigations into the effects of heat stress have included analyses of chloroplast ultrastructure. Zhang et al. (2010) determined that increased air temperatures induced chloroplast swelling in *Arabidopsis*. This swelling was coordinated with increased non-photochemical quenching (NPQ). Chloroplast swelling has also been linked to increased chloroplastic pH and grana destacking (Semenova, 2002). Chloroplast ultrastructure changes have also been observed in response to potassium deficiency (Zhao et al., 2001), arsenic (Li et al., 2006), salt stress (Fidalgo et al., 2004), nitrogen fertilization (Bondada and Syvertsen, 2003), drought (Vassileva et al., 2012), and heat (Vani et al., 2001; Xu et al., 2006; Zhang et al., 2010). Genotypic variation in chlorophyll a/b ratios has been reported for soybean, but structural or functional implications were not investigated (Fritschi and Ray, 2007). Surprisingly, to date, no investigations of genotypic differences in chloroplast ultrastructure have been reported for soybean.

The primary route for energy from absorbed light is its use for the light-dependent reactions of photosynthesis. Excess energy can be dissipated as heat, NPQ, and as light in the form of fluorescence. Chlorophyll fluorescence can be evaluated rapidly and nondestructively, and can be used to provide information on the efficiency of the light-dependent reactions (Maxwell and Johnson, 2000). The vast body of work that exists about chlorophyll fluorescence and its relationship to the light-dependent reactions has allowed calculations that reflect the state of various processes of the light-dependent reactions. Thus, chlorophyll fluorescence measurements have significantly enhanced our understanding of the effects of abiotic stresses on the biochemical steps of the light-dependent reactions of photosynthesis (Strasser and Strasser, 1995). Chlorophyll fluorescence measurements have been used to assess the effects of abiotic stress factors on the light-dependent reactions, including the effects of nitrogen deficiency (Congming and Jianhua, 2000; Huang et al., 2004), salt stress (Misra et al., 2001; Woo et al., 2008), and heat stress (Schreiber and Berry, 1977; Briantais et al., 1996; Zhou et al., 2018). Damage to PS2 caused by elevated temperature can be observed as increases in minimum fluorescence (Briantais et al., 1996) and reduced grana stacking (Yamamoto et al., 2008). Damage to PS2 can lead to destacking of the grana and alter ATP and NADPH production ratios. Reductions in NADPH caused by damage to PS2 can limit the availability of reducing power for Rubisco and thus change both CO_2_ assimilation and Rubisco activase repair and synthesis (Murata et al., 2007). Although potentially valuable to devise strategies to improve soybean heat tolerance, detailed physiological studies about light-dependent reaction responses of soybean to heat stress are lacking.

This study was conducted to determine the effects of elevated temperatures on chloroplast ultrastructure, chlorophyll fluorescence, and gas exchange of soybean. To this end, four genotypes were grown in growth chambers and three temperature regimes were imposed for 7 days after initial growth in uniform conditions. The effects of the different temperature regimes on chloroplast ultrastructure, chlorophyll fluorescence, and gas exchange were assessed. The study provides novel information about the effects of high temperatures on the structure and function of soybean chloroplasts which may be used for crop improvement efforts and for studies aimed at further dissection of heat stress tolerance mechanisms in soybean.

## Materials and Methods

### Genotypes and Growth Conditions

Four soybean genotypes were selected for inclusion in this experiment based on the characterization of 120 diverse maturity group (MG) IV soybean genotypes. Briefly, the soybean genotypes were grown under control conditions (28°C) in a greenhouse until at least the 4^th^ leaf stage after which they were exposed to elevated air temperature (> 40°C) on three non-consecutive days. Chlorophyll fluorescence measurements were conducted on each day of elevated air temperature. Two genotypes from this experiment were selected for inefficient (PI603166) and efficient (PI398226) photosynthetic parameters in elevated air temperature based on rankings of more than 20 chlorophyll fluorescence phenotypes. PI603166 was ranked in the bottom 5% whereas PI398226 was ranked in the top 5% based on chlorophyll fluorescence measurements. The remaining two genotypes, DS25-1 (tolerant) and DT97-4290 (sensitive), were included in the greenhouse screening and in the present study because they differ in germinability of seeds that developed under elevated temperature (Smith, 2017). Hereafter, genotypes will be referred to as PI-26, PI-66, DS25, and DT97 for PI398226, PI603166, DS25-1, and DT97-4290, respectively. Five seeds of each genotype were sown approximately 2.5 cm deep in each of six pots (12.7 cm × 12.7 cm × 30.5 cm) filled with top soil (Ri-Mor Mulch and Landscape, Columbia, MO, USA) placed in one of two PGR15 growth chambers (Controlled Environments Ltd, Winnipeg, MB, Canada). After plants had their 1^st^ unrolled trifoliate (V1; Fehr et al., 1971) pots were thinned to a single plant, aiming for uniform height and vegetative stage across pots. Growth chamber conditions were set to 14 h of light (1,000 µmol m^-2^ s^-1^ PAR) and 28°C and 50% relative humidity during the light hours (6 am to 8 pm) and 22°C and 75% relative humidity in the dark. Temperatures were increased from 22 to 28°C 1 h after lights were turned on and returned to 22 from 28°C 1 h after lights were turned off. Plants were grown for three additional weeks until they reached four fully expanded trifoliate leaves. At that time, three temperature treatments were initiated, namely 28, 38, and 45°C maximum air temperatures which were imposed as follows: For the control treatment (28°C), the temperature regime was maintained the same as for initial growth. For elevated air temperature treatments, the temperature was increased gradually from 28°C at 7:00 am to reach 38 or 45°C by 9:00 am and held at these temperatures until 1:00 pm. Then the temperature was reduced from 38 or 45°C to 28°C from 1:00 pm to 3:00 pm and maintained at 28°C until 8:00 pm. Elevated temperatures that occur during the growing season often come in the form of heat waves that generally last a few to several days. Therefore, temperature treatments were imposed over the course of 7 days, at which point the experiments were terminated.

Each temperature regime was imposed in two separate experiments, once in each of the two growth chambers that were used for this study. Chlorophyll fluorescence measurements and gas exchange measurements were collected both times while samples for microscopy were collected in one of the two experiments. For each experiment, the four genotypes were grown in six replications arranged as a completely randomized design.

### Chlorophyll Fluorescence and Net Photosynthesis Measurements

Chlorophyll fluorescence was measured every day during the 7 days of temperature treatment imposition, starting at 10:00 am (1 h after reaching maximum temperature in the 38 and 45°C treatments) and finishing at approximately 10:30 am. Leaf clips were attached to the center leaflet of the 2^nd^ fully expanded trifoliate leaf for 20 min of dark adaptation. Chlorophyll fluorescence measurements were obtained using the OJIP protocol of a Fluorpen Z995-PAR (Qubit systems INC, Kinston Ontario, Canada). Due to the number of phenotypes, time points and treatments, only a subset of phenotypes (F_0_, F_M_, F_V_/F_M_, and ABS/RC) and time points (days 1, 3, 5, and 7) that simplified but captured the differences and similarities observed in chlorophyll fluorescence, were selected to be presented here.

The same leaves that were measured for chlorophyll fluorescence were also used for net photosynthesis measurements with a portable photosynthesis system (LI-6400XT, LI-COR, Lincoln, NE, USA). The conditions in the measurement chamber were controlled at ambient CO_2_ level (380 µmol mol^-1^), leaf block temperature was set to match the air temperature of the respective treatment (28, 35, or 45°C), and photosynthetically active radiation was set to 1,000 µmol m^-2^ s^-1^ to match the light intensity in the growth chamber. Gas exchange and leaf temperatures were measured in each replication on the 1^st^, 3^rd^, 5^th^, and 7^th^ day of temperature treatment starting at approximately 10:30 am (~1.5 h after reaching maximum temperature in the 35 and 45°C treatments).

### Chloroplast Ultrastructure Assessment

Tissue samples for chloroplast ultrastructure analysis were obtained on the 1^st^ and 7^th^ day of the 28 and 45°C treatments from genotypes DT97 and PI-66 at approximately 12:30 pm after leaf gas exchange measurement before temperatures began changing. These two genotypes and the 28 and 45°C temperature treatments were selected for examination of chloroplast ultrastructure based on the chlorophyll fluorescence measurements conducted during the greenhouse screening. One leaf disk (1 mm diameter) was collected from the area of the center leaflet of the 2^nd^ fully expanded leaf that was used for chlorophyll fluorescence measurements and was immediately placed into primary fixative (5% glutaraldehyde). Samples were post fixed in osmium tetroxide (OsO_4_) and dehydrated in increasing concentrations of ethanol. Samples were embedded in Spurr’s epoxy and ultrathin sections were cut using a microtome set to 60 nm for the 28°C treatment and to 80 nm for the 45°C treatments (due to impaired integrity of the sections at 60 nm). All samples were post stained with Reynold’s Lead Citrate. Sections were viewed with a JEOL JEM-1400 transmission electron microscope (JEOL Ltd. Akishima, Tokyo, Japan). Images of chloroplasts were obtained at ×7,000 magnification. Multiple images were collected per chloroplast and were stitched to form one image of each chloroplast at ×7,000 using MS paint (Microsoft Corporation, Redmond, WA, USA). ImageJ (Schneider et al., 2012) was used to measure ultrastructural traits of chloroplasts using the 1 µm scale bar of the electron microscopic images to scale pixels. Twenty-five chloroplasts from each of the two temperature treatments and genotypes were analyzed for total grana number, total grana area, average grana area, total number of starch grains, total starch grain area, average starch grain area, chloroplast area, percent grana area, and percent starch grain area. To assess total grana number per chloroplast, each granum was counted for each of the chloroplasts and totaled. Total grana area was assessed by measuring the area of all grana by tracing lines around each grana of all chloroplasts and then summing the areas. Average grana area was calculated by dividing the total grana area by the number of grana for each chloroplast. Total number of starch grains was assessed by counting the number of starch grains present in each chloroplast. Total starch grain area was measured by tracing edges of starch grains and then adding all the areas together. Average starch grain area was calculated by dividing the total area of starch grains by the number of starch grains for each chloroplast. Chloroplast area was measured by tracing the outside of each chloroplast. Percent grana area was calculated by dividing the total grana area by the chloroplast area and multiplying by 100. Percent starch grain area was calculated by dividing the total starch grain area by chloroplast area and multiplying by 100.

### Statistical Analysis

Statistical analyses were performed with SAS 9.4 (SAS institute Inc. 2004). Repeated measures analysis was performed by PROC MIXED with genotype by treatment by experiment nested within sample as the repeated measure and the model genotype, day of treatment, temperature treatment, and experiment as main effects and including the respective interaction effects for chlorophyll fluorescence and gas exchange data. While significant differences were found between repeat experiments for some phenotypes, data trends across days of treatments were the same. Therefore, statistical analysis and data presentation were considered together. Analysis of variance for chloroplast ultrastructure was performed with PROC GLM by genotype with temperature treatment, day of treatment, replication, and respective interaction effects included in the model. Pair-wise Tukey analysis was used to identify differences between treatments by genotype and day of treatment for the chloroplast ultrastructure.

## Results

### Effects of Temperature Treatments on Gas Exchange

**Figure 1:**
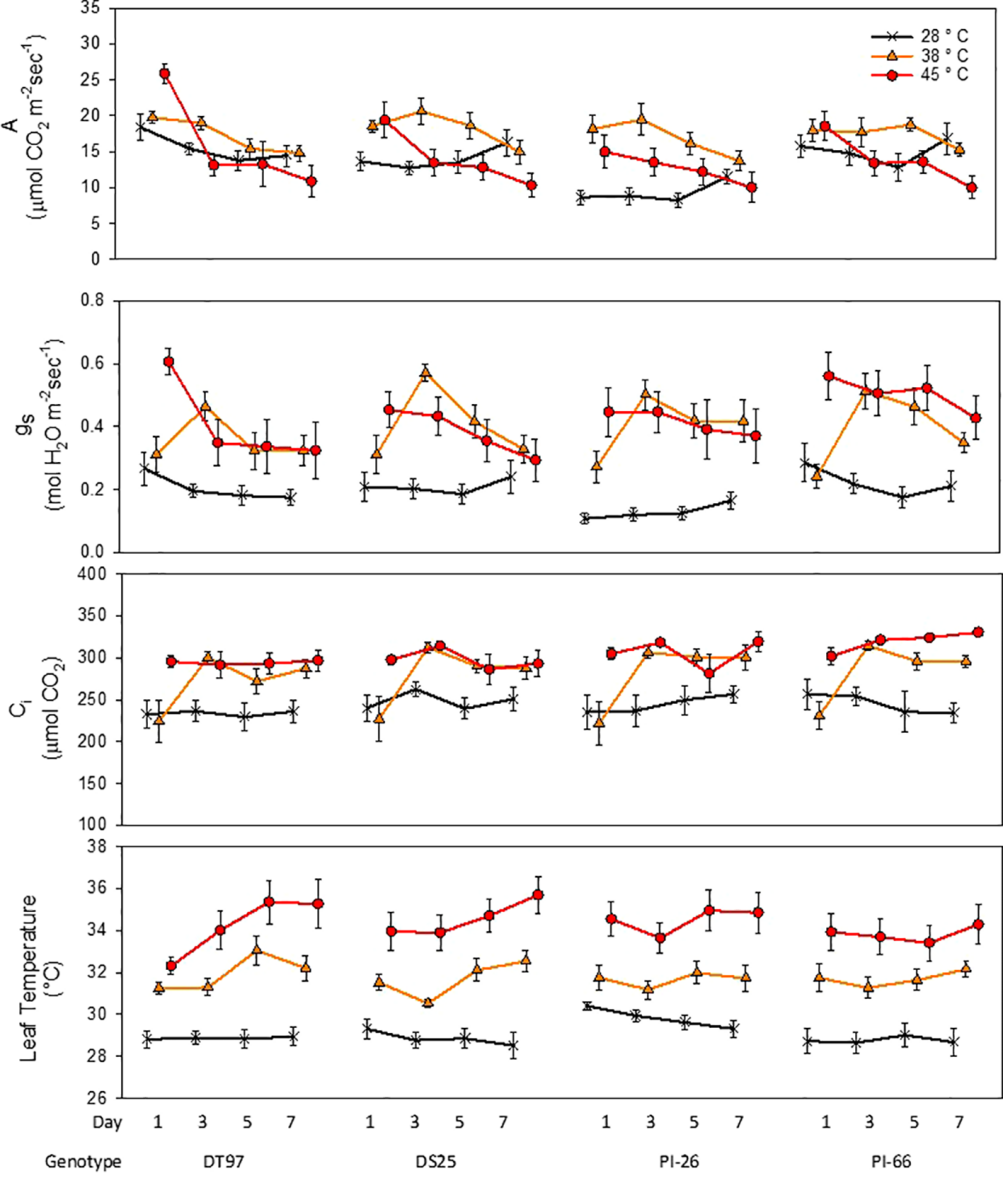
Responses of photosynthetic rate (A), stomatal conductance (g_s_), internal CO_2_ (C_i_), and leaf temperature of four soybean genotypes to the imposition of maximum day time temperatures of 28, 38, and 45°C over the course of 7 days. Measurements were taken from the uppermost middle trifoliate leaves 2 h after air temperatures reached the indicated temperature on days 1, 3, 5, and 7. Symbols represent mean values (N = 12) and whiskers indicate standard error.

Leaf net photosynthesis (A) was measured between 1.5 and 4 h after air temperatures in the growth chambers reached their respective treatment maxima on the 1^st^, 3^rd^, 5^th^, and 7^th^ day of temperature treatments (**Figure 1** and **Table 1**). In the 45°C treatment, A was greatest on the 1^st^ day of treatment and declined over the course of the 7-day treatment period in all four genotypes (**Figure 1**). In contrast, A in the control treatment (28°C) was more stable over the course of the 7 days, decreasing slightly in DT97 or increasing somewhat in DS25, PI-26, and PI-66. While A at 45 and 28°C did not differ on the 1^st^ day in PI-66, it was lower at 45°C on the 7^th^day. In contrast, A did not differ between 45 and 28°C in DT97, on the 1^st^ or 7^th^ days. Leaf net photosynthesis (A) of PI-26 at 28°C was lower than in the other three genotypes and started out lowest with 28 and 45°C treatments on the 1^st^ day but did not differ from 38 and 45°C treatments on the 7^th^ day. In general, A in the 38°C treatment was high in the beginning or even increased slightly early on and declined only after the 3^rd^ or 5^th^ day of treatment.

**Table 1:** Statistical groupings from **Figure 1** for photosynthetic rate (A), stomatal conductance (g_s_), internal CO_2_ (Ci) and leaf temperature.

Genotype	A	28	38	45	g_s_	28	38	45
DT97	1^st^ day	AB	B	A	1^st^ day	B	B	A
	3^rd^ day	B	A	AB	3^rd^ day	B	A	A
	5^th^ day	A	A	A	5^th^ day	B	A	AB
	7^th^ day	A	A	A	7^th^ day	B	A	A
DS25	1^st^ day	B	AB	A	1^st^ day	B	AB	A
	3^rd^ day	B	A	AB	3^rd^ day	B	A	A
	5^th^ day	AB	A	B	5^th^ day	B	A	AB
	7^th^ day	A	A	B	7^th^ day	B	A	AB
PI-26	1^st^ day	C	A	B	1^st^ day	B	A	A
	3^rd^ day	C	A	B	3^rd^ day	B	A	A
	5^th^ day	B	A	AB	5^th^ day	B	A	A
	7^th^ day	A	A	A	7^th^ day	B	A	A
PI-66	1^st^ day	A	A	A	1^st^ day	B	B	A
	3^rd^ day	AB	A	B	3^rd^ day	B	B	A
	5^th^ day	B	A	B	5^th^ day	B	B	A
	7^th^ day	A	AB	B	7^th^ day	B	AB	A
	**Ci**	**28**	**38**	**45**	**LTemp**	**28**	**38**	**45**
DT97	1^st^ day	AB	B	A	1^st^ day	B	A	A
	3^rd^ day	B	A	A	3^rd^ day	B	AB	A
	5^th^ day	A	A	A	5^th^ day	C	B	A
	7^th^ day	B	A	A	7^th^ day	C	B	A
DS25	1^st^ day	A	A	A	1^st^ day	B	A	A
	3^rd^ day	A	A	A	3^rd^ day	C	B	A
	5^th^ day	A	A	A	5^th^ day	C	B	A
	7^th^ day	B	A	AB	7^th^ day	C	B	A
PI-26	1^st^ day	B	B	A	1^st^ day	B	A	A
	3^rd^ day	B	B	A	3^rd^ day	B	A	A
	5^th^ day	A	A	A	5^th^ day	C	B	A
	7^th^ day	B	A	A	7^th^ day	C	B	A
PI-66	1^st^ day	B	AB	A	1^st^ day	B	A	A
	3^rd^ day	B	A	A	3^rd^ day	C	B	A
	5^th^ day	B	AB	A	5^th^ day	B	A	A
	7^th^ day	B	A	A	7^th^ day	B	A	A

Same letter represents temperature treatments that were grouped together based on differences of least square means.

In general, stomatal conductance (g_s_) followed similar temporal patterns for the different temperature treatments as the responses of A (**Figure 1**). Stomatal conductance was lowest and more stable across the 7 days in the control treatment compared to the 38 and 45°C treatments. In the 45°C treatment g_s_ decreased from the 1^st^ to 7^th^ day in all genotypes. In contrast, at 38°C, g_s_ of all genotypes increased sharply from the 1^st^ to the 3^rd^ day of treatment, followed by a steady decline over the course of the remaining days with DS25 and PI-66. On average across the 7-day treatment period, g_s_ at 28°C was lower in PI-26 than in the other three genotypes which had similar g_s_. This lower gs of PI-26 was consistent with the lower A observed for PI-26 in the control treatment.

Internal CO_2_ concentration (C_i_) in the 28 and 38°C treatments followed a similar temporal pattern as g_s_ across the 7-days for all genotypes (**Figure 1** and **Table 1**). However, C_i_ in the 45°C treatment was more stable than g_s_ and A did not exhibit a pronounced decline over the course of the heat treatment. In fact, in PI-66, a small increase in C_i_ occurred with the 45°C treatment over the course of the heat treatment period.

Treatments strongly influenced leaf temperatures of all genotypes (**Figure 1**). Leaf temperatures were relatively stable across the 7 days in the 28°C treatment in DT97, DS25, and PI-66. However, a small decline (1.2°C decrease) of leaf temperature from the 1^st^ to 7^th^ day was observed for PI-26 in the 28°C treatment. In the 38 and 45°C treatments, leaf temperatures of PI-26 (maximum difference among days ~1.3°C) and PI-66 (maximum difference among days <0.9°C) were relatively stable. While leaf temperatures in the 38°C of DT97 increased from the 3^rd^ to 5^th^ day, and a steady increase of leaf temperature with the 45°C treatment. A small decrease in leaf temperature on the 3^rd^ day of 38°C was observed with DS25 followed by increasing leaf temperatures on the 5^th^ and 7^th^ days.

### Temperature Treatment Influences on Chlorophyll Fluorescence

**Figure 2:**
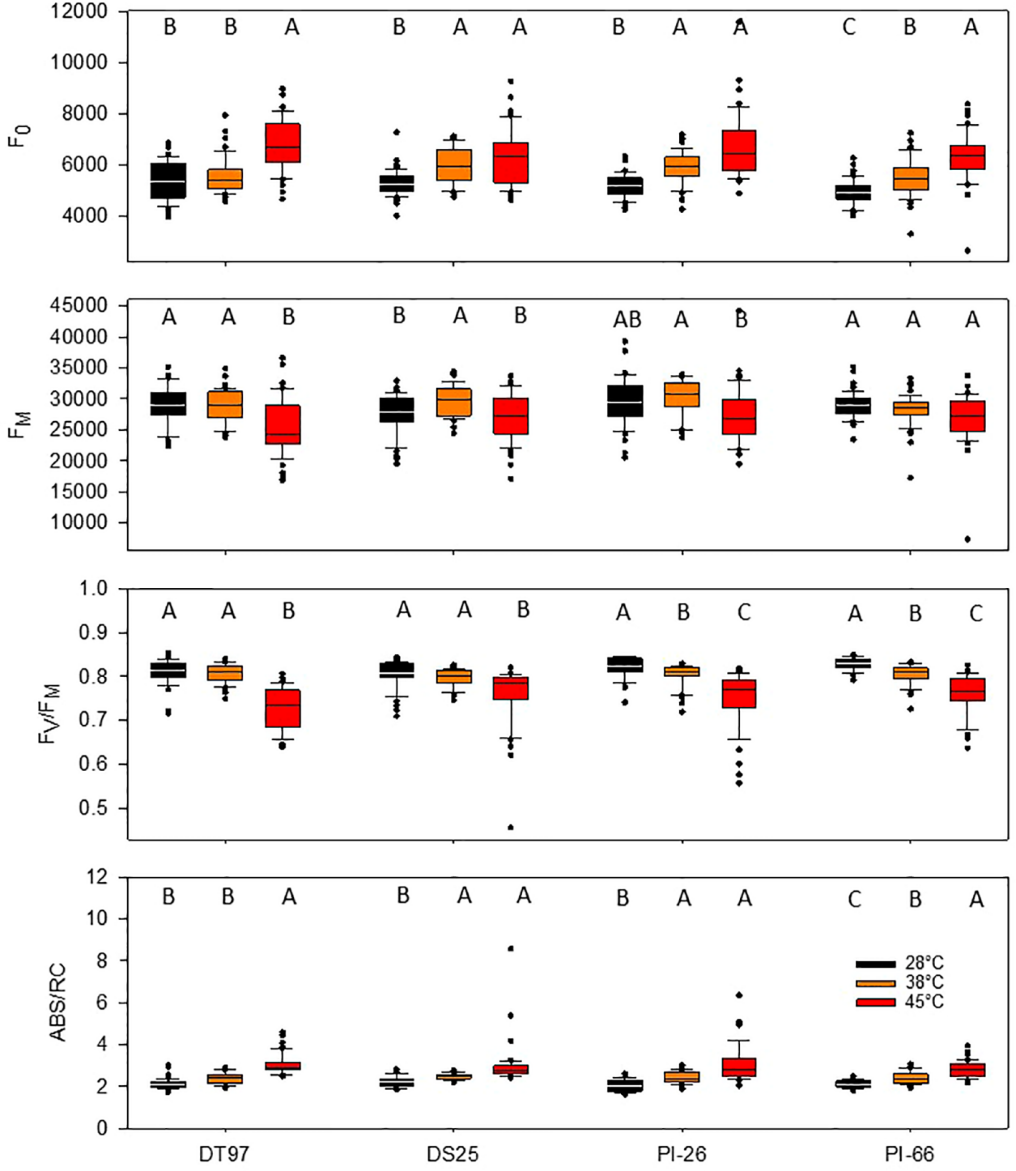
Minimum fluorescence (F_0_), maximum fluorescence (F_M_), maximum photochemical efficiency (F_V_/F_M_), and absorption per reaction center (ABS/RC) of four soybean genotypes exposed to maximum day time temperatures of 28, 38, and 45°C over the course of 7 days. Data represents chlorophyll fluorescence measurements on the center leaflet of the upper-most fully expanded leaf after 1 h of temperature treatment on days 1, 3, 5, and 7 from two independent growth chamber experiments for each temperature treatment (N = 48). Lines within boxes represent the median and top and bottom of whiskers represent the 1^st^ and 3^rd^ quartiles respectively. Circles represent outlier data one and a half times the 1^st^ and 3^rd^ quartiles. Letters indicate similar treatments of phenotypes within genotype based on Tukey pairwise analysis.

**Figure 3:**
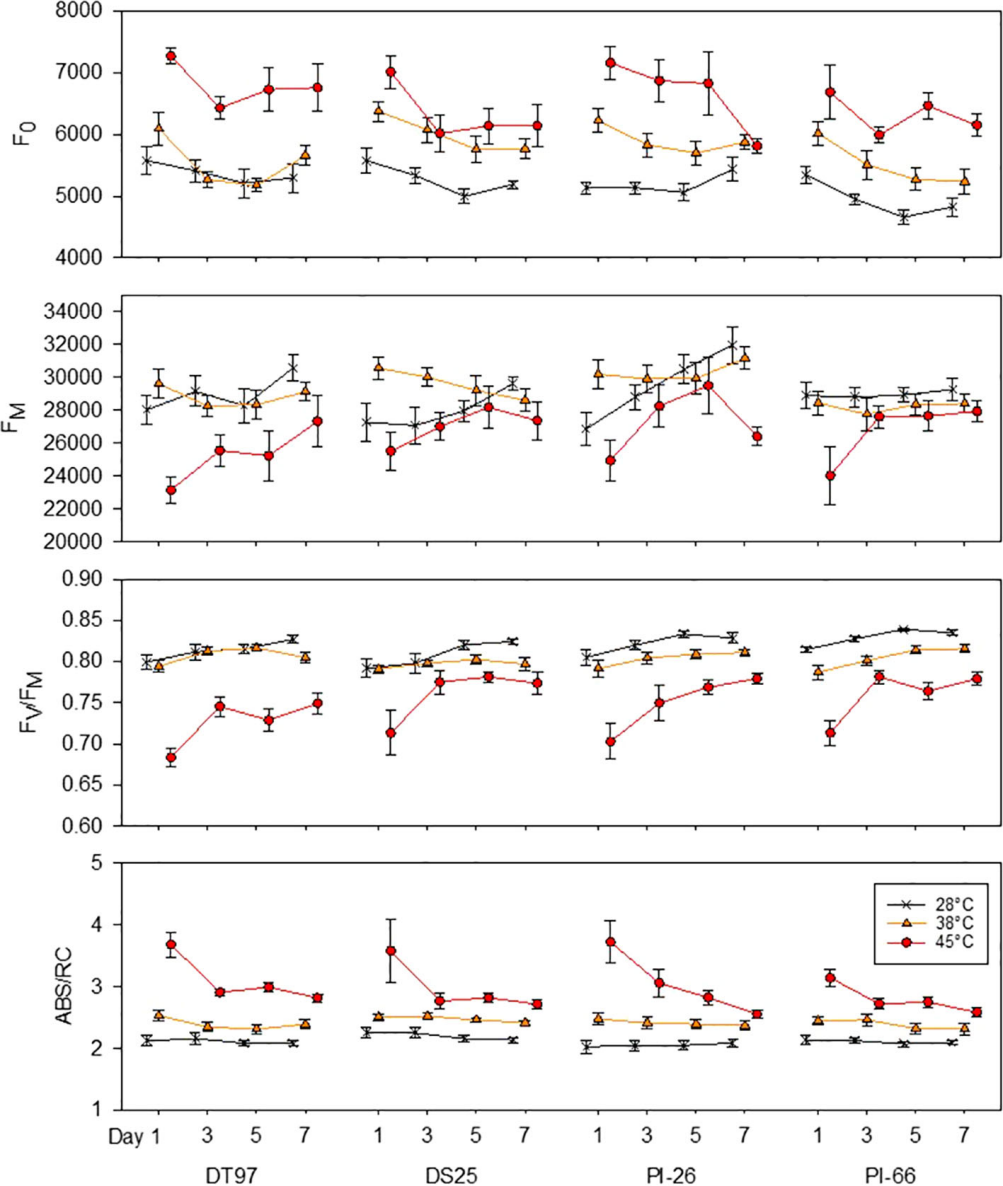
Responses of minimum fluorescence (F_0_), maximum fluorescence (F_M_), maximum photochemical efficiency (F_V_/F_M_), and absorption per reaction center (ABS/RC) of four soybean genotypes to the imposition of maximum day time temperatures of 28, 38, and 45°C over the course of 7 days. Measurements weretaken from the middle leafleft of the uppermost fully expanded leaf between one and two hours after air temperatures reached the indicated temperature on days 1, 3, 5, and 7. Symbols represent mean values (N = 12) and whiskers indicate standard error.

Minimum fluorescence (F_0_) provides information on the status of PS2 which is critical for the overall efficiency of the light reactions. Analysis of F_0_ by treatment across all 7 days revealed that F_0_ increased in all genotypes when exposed to 45°C as compared to the 28°C treatment (**Figure 2**). In DT97, PI-26, and PI-66, F_0_ at 38°C was lower than at 45°C and higher than in the 28°C treatment, whereas, in DT97, F_0_ at 38°C was similar to the 28°C treatment. Temporal analysis of F_0_ indicated that F_0_ was higher at 45°C than at 28°C throughout the 7-day heat treatment in DT97 and PI-66, while in DS25 and PI-26, it was higher on all but the last day of measurements (**Figure 3** and **Table 2**). In the 38°C treatment, F_0_ tended to be intermediate in PI-26 and PI-66 or similar to the 45°C treatment in DS25 for much of the 7-day treatment period or similar to the 28°C treatment in DT97. The temporal pattern of F_0_ in the different temperature treatments generally were similar within a genotype, except for a dramatic drop on the 7^th^ day in PI-26 grown at 45°C.

**Table 2:** Statistical groupings for traits from **Figure 3**.

Genotype	F_0_	28	38	45	F_M_	28	38	45
DT97	1^st^ day	B	B	A	1^st^ day	A	A	B
	3^rd^ day	B	B	A	3^rd^ day	A	A	B
	5^th^ day	B	B	A	5^th^ day	A	A	B
	7^th^ day	B	B	A	7^th^ day	A	AB	B
DS25	1^st^ day	C	B	A	1^st^ day	B	A	B
	3^rd^ day	B	A	A	3^rd^ day	B	A	B
	5^th^ day	B	A	A	5^th^ day	A	A	A
	7^th^ day	B	AB	A	7^th^ day	A	AB	B
PI-26	1^st^ day	C	B	A	1^st^ day	B	A	B
	3^rd^ day	C	B	A	3^rd^ day	A	A	A
	5^th^ day	C	B	A	5^th^ day	A	A	A
	7^th^ day	A	A	A	7^th^ day	A	A	B
PI-66	1^st^ day	C	B	A	1^st^ day	A	A	B
	3^rd^ day	B	AB	A	3^rd^ day	A	A	A
	5^th^ day	C	B	A	5^th^ day	A	A	A
	7^th^ day	B	B	A	7^th^ day	A	A	A
	**F_V_/F_M_**	**28**	**38**	**45**	**ABS/RC**	**28**	**38**	**45**
DT97	1^st^ day	A	A	B	1^st^ day	C	B	A
	3^rd^ day	A	A	B	3^rd^ day	B	B	A
	5^th^ day	A	A	B	5^th^ day	B	B	A
	7^th^ day	A	A	B	7^th^ day	B	B	A
DS25	1^st^ day	A	A	B	1^st^ day	B	B	A
	3^rd^ day	A	A	A	3^rd^ day	B	AB	A
	5^th^ day	A	AB	B	5^th^ day	B	B	A
	7^th^ day	A	B	B	7^th^ day	B	AB	A
PI-26	1^st^ day	A	A	B	1^st^ day	C	B	A
	3^rd^ day	A	A	B	3^rd^ day	C	B	A
	5^th^ day	A	A	B	5^th^ day	C	B	A
	7^th^ day	A	A	B	7^th^ day	B	AB	A
PI-66	1^st^ day	A	B	C	1^st^ day	B	B	A
	3^rd^ day	A	B	B	3^rd^ day	B	A	A
	5^th^ day	A	A	B	5^th^ day	B	B	A
	7^th^ day	A	A	B	7^th^ day	B	AB	A

Same letter represents temperature treatments that were grouped together based on differences of least square means.

The capacity for energy utilization by the light-dependent reactions can be assessed with maximum fluorescence (F_M_). Across the 7 days of treatment, maximum fluorescence of DT97, PI-26, and PI-66 in the 45°C treatment was lower than in the 28 and 38°C treatments (**Figure 2**). However, the F_M_ of DS25 was lower in the 28 and 45°C treatments than the 38°C treatment. Maximum fluorescence was relatively consistent throughout the 7-day period in the 28 and 38°C treatments in DT97 and PI-66 (**Figure 3**). In the 45°C treatment, F_M_ generally varied more and was greater on the 7^th^ day compared to the 28 and 45°C treatments in PI-26. While F_M_ at 45°C was maximal on the 7^th^ day in DT97, F_M_ maxima occurred on day 5 in DS25 and PI-26, and in PI-66 a sharp increase was observed from day 1 to day 3 after which it remained steady.

Photochemical efficiency (F_V_/F_M_) is a measure of the maximum photosystem II efficiency. In general, F_V_/F_M_ declined with increasing temperature in all four genotypes (**Figure 2**). In PI-26 and PI-66 the decrease in F_V_/F_M_ was significant between every temperature regime, whereas in DT97, F_V_/F_M_ was lower at 45°C than at both 28 and 38°C, which were not different from each other. On average, across all days and compared with F_V_/F_M_ at 28°C, F_V_/F_M_ at 45°C was reduced by 10.3, 5.39, 8.53, and 7.86%, in DT97, DS25, PI-26, and PI-66, respectively. Over the course of the 7-day treatments, a considerable increase in F_V_/F_M_ was observed for all genotypes at 45°C whereas it increased only slightly or was relatively stable at 28 and 38°C. In DT97, DS25, and PI-66, increases in F_V_/F_M_ from the 1^st^ to the 3^rd^ day were observed at 45°C, but no change was found after that. In contrast, a more gradual increase in F_V_/F_M_ from 1^st^ to 7^th^ day was observed in PI-26 in 45°C treatment. The F_V_/F_M_ in 45°C recovered from 85.5% of the control (28°C) on day 1 to 90.6% of the control on the 7^th^ day in DT97, and from 90.1 to 93.9%, 87.3 to 94.1%, and 87.6 to 93.3% in DS25, PI-26, and PI-66, respectively.

The absorption per reaction center (ABS/RC) represents the total number of photons absorbed by chlorophyll molecules in proportion to the total number of active reaction centers. Across all measurement days, all genotypes showed an increase in ABS/RC with increasing temperature (**Figure 2**). Over the course of the 7 days, ABS/RC in the 28 and 38°C treatments were relatively stable and differed little between these two treatments (**Figure 3** and **Table 2**). On the other hand, ABS/RC at 45°C was greater than that at 28°C in all genotypes and on all measurement days. On the 1^st^ day, ABS/RC at 45°C was dramatically greater than at 38°C in all genotypes. However, by the 7^th^ day ABS/RC had declined in the 45°C treatment such that differences between 38 and 48°C were not significant anymore in DS25, PI-26, and PI-66.

### Effects of Heat Stress on Chloroplast Ultrastructure

**Figure 4:**
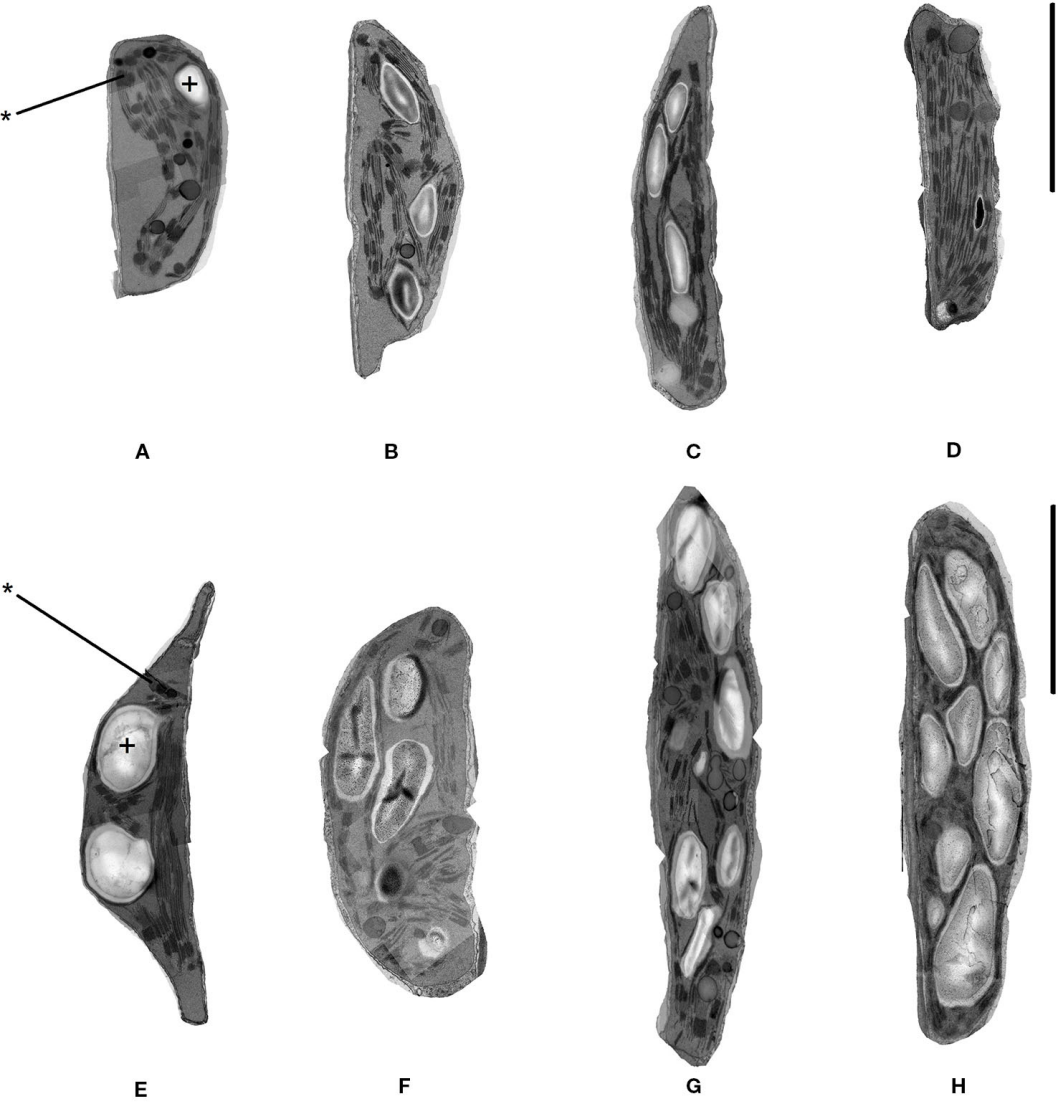
Representative transmission electron microscope images of chloroplasts in upper-most fully expanded trifoliate leaves from DT97 **(A**–**D)** and PI-66 **(E**–**H)**. Images were acquired from samples collected on the 1^st^ day of 28°C treatment **(A, E)**, 7^th^ day of 28°C treatment **(B, F)**, 1^st^ day of 45°C treatment **(C, G)**, and 7^th^ day of 45°C treatment **(D, H)**. Black bars represent 1.0 µm. Starch grains and grana are identified with + and *, respectively.

**Figure 5:**
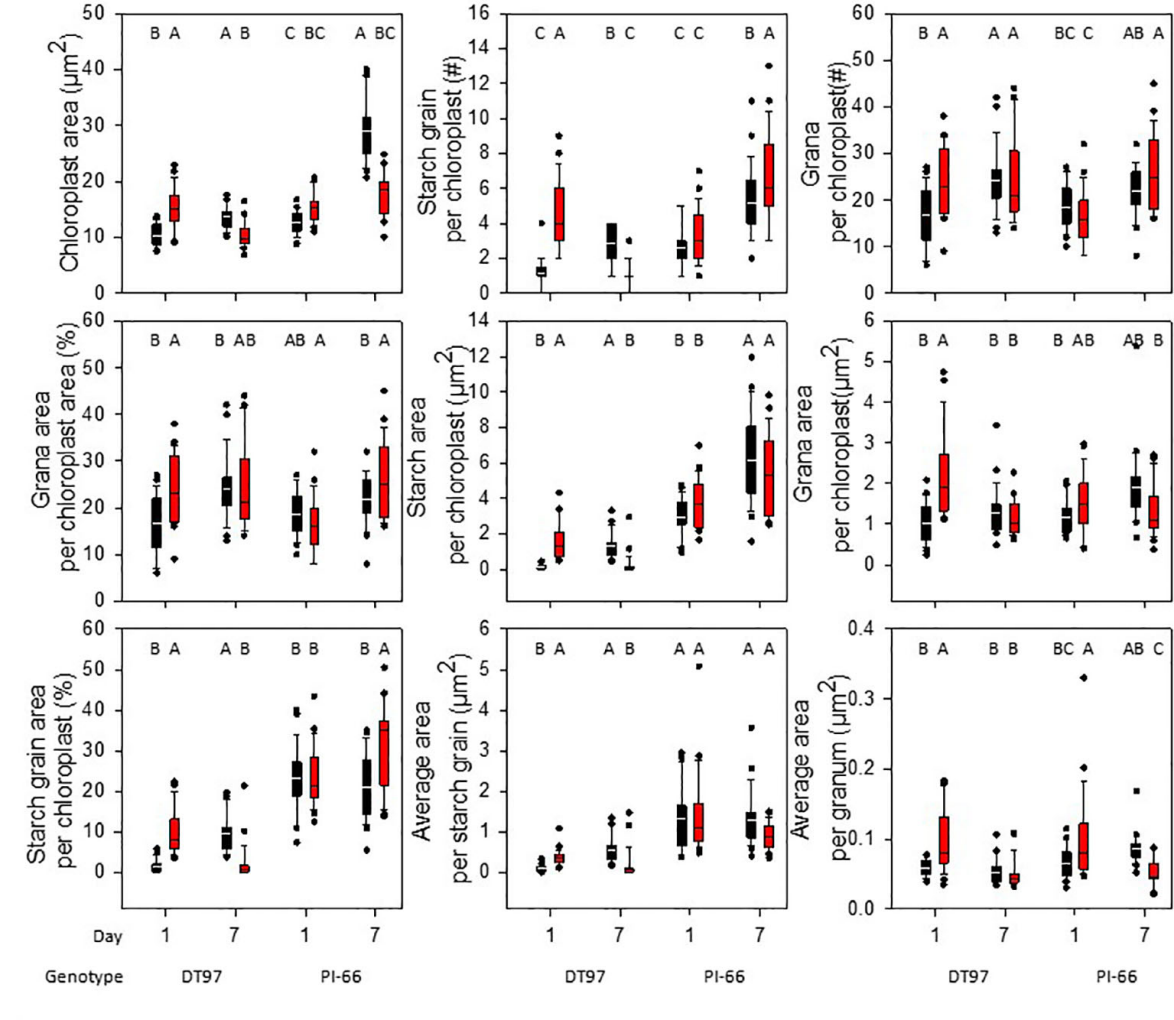
Chloroplast ultrastructure characteristics in upper-most fully expanded leaves of DT97 and PI-66 from the 1^st^ and 7^th^ day of 28 and 45°C temperature treatments. Traits were assessed on transmission electron microscopy images of 25 chloroplasts for each time point and temperature treatment of both genotypes (N = 25). Lines within boxes represent the median and top and bottom of whiskers represent the 1^st^ and 3^rd^ quartiles respectively. Circles represent outlier data one and a half times the 1^st^ and 3^rd^ quartiles. Letters indicate similar treatments of phenotypes within genotype based on Tukey pairwise analysis.

Chloroplast ultrastructure was examined in leaf tissues sampled after gas exchange measurements on the 1st and 7^th^ days from DT97 and PI-66 plants grown at 28 and 45°C. Representative transmission electron micrographs depicted in **Figure 4** illustrate genotype differences at both 28 and 45°C as well as heat treatment effects on both genotypes. Quantitative assessment of chloroplast characteristics based on transmission electron microscopy (TEM) images revealed that the responses to 7 days of 28 or 45°C differed between genotypes for chloroplast area, percent starch grain area per chloroplast, starch area per chloroplast, and average area per granum (**Figure 5**). On the 1^st^day, the chloroplast area was greater at 45°C than at 28°C in DT97 but similar in PI-66. On the 1^st^ day at 45°C, the number of starch grains and grana per chloroplast were greater than at 28°C in DT97. In contrast, no treatment effect on starch grain and grana numbers per chloroplast were found in PI-66 after the 1^st^ day. Both percent grana area per chloroplast as well as grana area per chloroplast was greater at 45°C than at 28°C in DT97 on the 1^st^ day but did not differ on the 7^th^ day. In PI-66, no differences in the percent grana area per chloroplast were observed on either day, but the grana area per chloroplast was larger at 45°C than 28°C on the 1^st^ day and smaller at 45°C than at 28°C on the 7^th^ day. As can be seen in **Figures 4** and **5**, the starch area per chloroplast was greater in the heat stress treatment on the 1^st^ day for DT97 but smaller than in the control by the 7^th^ day. In contrast, the starch area per chloroplast in PI-66 was not affected by temperature on the 1^st^ or 7^th^ day. However, the percent starch area per chloroplast was increased in PI-66 but decreased in DT97 in the 45°C compared to the 28°C treatment on the 7^th^ day. Overall, the starch was much less in DT97 than PI-66, and this was associated with smaller starch grains of DT97 than PI-66. In response to 45°C compared to 28°C, DT97 exhibited larger starch grains on the 1^st^ day, but smaller starch grains on the 7^th^ day. Unlike in DT97, the size of individual starch grains was not influenced by the temperature treatment in PI-66. These results for average starch grain area paralleled those observed for the starch area per chloroplast.

## Discussion

### Temperature Treatment Effects on Leaf Temperatures and Gas Exchange Parameters

For the 7 days of temperature treatment imposition, air temperatures in the growth chambers were ramped up from 22°C (night time) over the course of 2 h to reach maximum daytime temperatures of 28 (control), 38, or 45°C by 9 am. These maximum temperatures were maintained for 4 h after which air temperatures were gradually reduced to the control temperature for the remaining time of the 14-h photoperiod, and then to night time temperature. Because plants were well-watered throughout the experiments, the imposition of these air temperature treatments, coupled with the control of relative humidity at 50% during the photoperiod, allowed for significant transpirational cooling which resulted in average leaf temperatures of 29.1, 31.9, and 34.5°C in the 28, 38, and 45°C treatments, respectively (**Figure 1**). Thus, the responses of gas exchange, chlorophyll fluorescence, and chloroplast ultrastructure of the genotypes to the three temperature treatments should be considered in the context of leaf temperatures and not only with respect to air temperatures.

In general, net photosynthetic rates observed in this study (18 µmol m^-2^ s^-1^—average across all genotypes) on the 1^st^ day of 28°C were comparable with those documented by others for growth chamber experiments at the same or similar temperature. For instance, Djanaguiraman et al. (2011) reported net photosynthetic rates of ~22 µmol m^-2^ s^-1^ for soybean genotype K 03-2897 grown at 28°C, and Ziska and Bunce (1995) reported ~12 µmol m^-2^ s^-1^ 18 days after emergence for the cultivar Clark grown at 25°C. Djanaguiraman et al. (2011) also exposed K 03-2897 to 38°C, and on the 2^nd^ and 6^th^ day measured net photosynthetic rates of ~20 and 17 µmol m^-2^ s^-1^, respectively. These values were similar to the net photosynthetic rates obtained in the present study on day 3 (~19 µmol m^-2^ s^-1^) and 7^th^ day (~17 µmol m^-2^ s^-1^), but were considerably lower than those normally observed under well-watered field conditions which generally are above 25 µmol m^-2^ s^-1^ (Haile and Higley, 2003; He et al., 2016; Yao et al., 2017; Sakowska et al., 2018). A decline of net photosynthesis is commonly observed in crops in response to increased temperatures. Reduced net photosynthesis in response to elevated temperatures has been shown for cotton (*Gossypium* spp.) (Salvucci and Crafts-Brandner, 2004) and soybean (Harley et al., 1985; Djanaguiraman et al., 2011). At elevated day/night temperatures of 48/28°C, photosynthesis in greenhouse-grown soybean was reduced from 24.9 µmol m^-2^ s^-1^ at 28/22°C day/night temperatures to 16.0 µmol m^-2^ s^-1^ (Jumrani et al., 2017). Similarly, soybean exposed to multiple short term (4 day) heat waves reduced photosynthetic rate of field grown soybean from ~36 µmol m^-2^ s^-1^ to ~20 µmol m^-2^ s^-1^ on the 1^st^ day of the 3^rd^ heat wave (Siebers et al., 2015). Similar to these studies, net photosynthesis was at elevated temperature. On average, across the 7-day treatment, net photosynthetic rates were greater (17.5 µmol m^-2^ s^-1^) at 38°C than at 45°C (14.0 µmol m^-2^ s^-1^) and 28°C (13.3 µmol m^-2^ s^-1^). However, these average values mask distinct temporal responses among the three treatments as well as differences among the genotypes.

At 45°C a significant decline in net photosynthesis was observed over the course of the 7 days of treatment. On the 1^st^ day of treatment, net photosynthesis at 45°C was greater than 28°C in DS25 and PI-26, but the decline was dramatic just as found for the other two genotypes. Similarly, while initially greater at 38°C than at 28°C in DS25 and PI-26, net photosynthesis at 38°C declined after 3 or 5 days and was not different from that in 28°C in any of the genotypes on 7^th^ day. Declines in photosynthesis with exposure to elevated temperatures (starting around 35°C) over the course of several days have been reported for other crop species (Crafts-Brandner and Salvucci, 2002; Salvucci and Crafts-Brandner, 2004). These findings may explain the high net photosynthetic rates and the initial lack of response observed in the 38°C treatment in the present study as plants were able to maintain leaf temperatures at approximately 31°C. Thus, transpirational cooling allowed soybean plants to maintain leaf temperatures in the range of 27–37°C when grown at 38°C air temperature, which resulted in similar or greater net photosynthetic rates than when grown at 28°C and leaf temperatures in the range of 25–32°C. Additionally, it is important to note that gas exchange measurements were conducted starting approximately 1.5 h after maximum temperatures were reached. Thus, it appears that, in conjunction with the transpirational cooling, the duration of exposure to 45°C on day 1 was not long enough to significantly impair net photosynthesis and that these soybean genotypes have the ability to tolerate exposures to such high air temperatures in the short term.

The response of stomatal conductance (g_s_) in the 38°C treatment differed from that in 45°C treatment such that g_s_ on the 1^st^ day was much greater at 45°C than at 28°C but similar to that at 28°C in the 38°C treatment. However, g_s_ increased dramatically from day 1 to day 3 in the 38°C treatment, to levels similar or greater than those observed in the 45°C treatment in all genotypes. This strong response suggests that day 1 exposure to 38°C preconditioned subsequent stomatal regulation. Similar responses of g_s_ to increased temperature have been shown at an even shorter time scale of under one hour (Hamerlynck and Knapp, 1996). It is likely that the dramatic response in g_s_ already occurred by day 2 of the treatment, but that remains to be shown.

Despite the low light environment, the photosynthetic rate of genotype PI-66 on the 7^th^ day of the 45°C treatment was reduced compared to the 28 and 38°C treatments and was in concurrence with the accumulation of starch in chloroplasts as shown by TEM. Conversely, the lower starch accumulation by DT97 on the 7^th^ day of the 45°C treatment compared to the control was associated with similar levels of photosynthetic rate between the 28 and 45°C treatments. Because of the importance of feedback mechanisms in photosynthetic regulation, determination of the effects of heat stress on the expression of photosynthetic genes and enzyme activities are important to advance mechanistic understanding of heat stress effects on photosynthesis in soybean.

### Chlorophyll Fluorescence Responses to Elevated Temperature Stress

Chlorophyll fluorescence measurements have been used broadly to elucidate the effects of abiotic stress factors on photosynthetic light reactions. In the present study, chlorophyll fluorescence measurements were made on the 1^st^, 3^rd^, 5^th^, and 7^th^ day of 7-day heat wave simulation treatments with maximum air temperatures of 38 and 45°C compared to a 28°C control treatment. Measurements of F_0_, F_M_, F_V_/F_M_, and ABS/RC revealed significant temperature treatment effects in all four soybean genotypes either throughout the heat stress period or at one of the four measurement days (**Figure 2** and **Figure 3**). On average across the duration of the heat wave, F_0_ was significantly greater in all four genotypes when they were exposed to 45°C compared to 28°C air temperatures, and in all genotypes but DT97, F_0_was also greater in the 38°C treatment than in the control (**Figure 2**). Temporal F_0_ dynamics illustrated in **Figure 3** and **Table 1** indicate differences among genotypes in acclimation over the course of the heat wave. Among the four genotypes, F_0_ in the 45°C treatment reached control levels only for PI-26 and only by 7^th^ day. It is well known that F_0_ increases in response to elevated temperature (Briantais et al., 1996; Yamane et al., 1997). Increases of F_0_ have been shown previously to occur in soybean in response to high light (Hong and Xu, 1999). Previous investigations of chlorophyll fluorescence in soybean in response to various abiotic stresses primarily reported on F_V_/F_M_ (Kao et al., 2003; Van Heerden et al., 2004; Ohashi et al., 2006). Increased F_0_ is caused by photoinhibitory damage to photosystem II (PS2) which has been shown to occur at elevated temperatures (Yamane et al., 1997). Impairment of PS2 occurs through oxidative damage to the D1 protein and subsequent removal and replacement of D1 are essential to maintaining efficient light-dependent reactions (Aro et al., 1993). Photoinhibition is influenced by the repair capacity of PS2, light harvesting antenna size, (Tyystjarvi et al., 1992), cyclic electron flow (Takahashi et al., 2009), NPQ (Niyogi et al., 1998), and D1 protein turn over (Aro et al., 1993). Indeed, cyclic electron flow (Bukhov and Carpentier, 2000; Egorova and Bukhov, 2002), NPQ (Sinsawat et al., 2004; Zhang et al., 2010), and D1 protein turn over (Yoshioka et al., 2006) have been shown to increase at elevated temperatures. Thus, it is possible that the higher temperature required to produce a response of F_0_ with DT97 to a temperature increase was due to increased repair capacity, and/or NPQ. This response of F_0_ in DT97 was paralleled by reduced net photosynthetic rates of the 38°C treatment that were similar to the 28°C treatment and on the 5^th^ and 7^th^ day the 45°C treatment had similar A to the 28 and 38°C treatments. Additional physiological studies will be needed to determine how DT97 is responding to increased temperature with respect to photoinhibition and implications for CO_2_ assimilation.

Maximum fluorescence (F_M_) is related with the donor side capacity of PS2, and has been shown to decrease in response to heat stress (Pospíšil et al., 1998; Mathur et al., 2011). Thus, the reduced F_M_ observed in this study, particularly early during heat stress imposition (**Figure 3**), is consistent with previous reports and a reduction of donor side capacity of PS2. The overall response of DT97 was similar to that of F_0_ in that an increase in temperature from 28 to 38°C did not have a profound effect, while a substantial impact was observed in response to the 45°C treatment. In contrast, unlike F_0_, an increase in temperature from 28 to 38°C did not have any effects on F_M_ in DT97, PI-26, and PI-66 and only an increase in temperature to 45°C lead to significant reductions in F_M_. In DS25, on average across days, an increase in temperature from 28 to 38°C lead to an increase in F_M_, but no difference was observed at 45°C compared to 28°C. The increase of F_M_ in DS25 was due to high F_M_ levels on the 1^st^ and 3^rd^ day and a return to the level of the 28°C treatment by the 5^th^ day. Temporal dynamics of F_M_ were more varied among the genotypes compared to F_0_. The F_M_ of PI-66 in the 45°C treatment rapidly recovered (by the 3^rd^ day) to the levels observed at 28°C, but F_M_ in DT97 recovered more slowly, never completely reaching those in the 28°C treatment. Overall, the temporal responses of F_M_ and F_0_ in the four genotypes indicate genotypic variation in the effects of high temperatures on the factors driving F_0_ compared to those underlying F_M_. Petkova et al. (2007), suggested that decreases in F_M_ observed in common bean cultivars (*Phaseolus vulgaris* L.) exposed to 42°C temperatures were due to an increase in NPQ. This suggestion seems to hold true for PI-66 given the observed responses of F_0_ and F_M_ to increased temperature. That is, F_M_ was relatively stable over the temperature treatments and was contrasted by increased F_0_. Thus, the capacity for protection against photoinhibition associated with NPQ may be reduced with PI-66 resulting in photoinhibition and increased F_0_. In the short term this reduced protection of PI-66 yielded similar photosynthetic rates between the 28 and 45°C treatments. However, on the 7^th^ day net photosynthetic rate at 45°C was lower than at 28 and 38°C. Reduced NPQ may have allowed more energy to be used by PI-66 in the 3^rd^ and 5^th^ days, while eventually causing increased damage resulting in reduced CO_2_ assimilation on 7^th^ day. Measurements of NPQ in PI-66 exposed to different temperatures will be needed to ascertain if NPQ is not responding to increased temperature in this genotype and how this relates to net photosynthesis.

Only DT97 and DS25 were able to maintain similar levels of F_V_/F_M_ in 38 and 28°C treatments (**Figure 2**). Genotypes PI-26 and PI-66 both exhibited reductions in F_V_/F_M_ in response to a temperature increase from 28 to 38°C. All genotypes exhibited reductions in F_V_/F_M_ with an increase in temperature from 38 to 45°C. Camejo et al. (2005), reported that the F_V_/F_M_ of wild tomato genotypes were similar under control (28°C) and heat (45°C) conditions while the increased temperature resulted in lower F_V_/F_M_ in a cultivar. Both F_V_/F_M_ and F_0_ are reliable indicators of photoinhibition due to heat stress (Gamon and Pearcy, 1989), as well as of high light (Ogren and Sjostrom, 1990), water stress (Epron et al., 1992), and low temperature (Groom and Baker, 1992). Thus, compared to the other two genotypes which exhibited reductions in F_V_/F_M_ in response to a temperature increase from 28 to 38°C, it appears that DT97 and DS25 can adapt to a 10°C increase. This was also supported by the net photosynthetic rates of DT97 which were similar at 45 and 28°C. Given the wealth of knowledge surrounding F_V_/F_M_ and the F_V_/F_M_ results for DT97 and DS25 documented here, soybean heat stress research and breeding may benefit from these two genotypes. To this end, DT97 may be of particular interest due to its ability to maintain lower levels of photoinhibition at higher temperatures compared to the other genotypes.

Absorption per reaction center (ABS/RC) represents the photon flux per reaction center. Overall, ABS/RC of all four genotypes increased with temperature. Temporal dynamics of ABS/RC at 45°C revealed rapid return to control or near-control levels in all genotypes except PI-26. The ABS/RC of PI-26 remained elevated compared to the other treatments until the 7^th^ day when the level of the 45°C treatment was similar to the 38°C but not the 28°C treatment. Absorption per reaction center is strongly driven, by the number of active reaction centers. Increases of ABS/RC have been shown to occur in soybean exposed to elevated temperatures (De Ronde et al., 2004), high salt stress in salt-stressed wheat (*T. aestivum*) (Mehta et al., 2010), and in nitrogen deficient corn and tomato (Kalaji et al., 2014). In contrast, ABS/RC was reduced in drought-stressed rice (Redillas et al., 2011). De Ronde et al. (2004), attributed the observed increase in ABS/RC in response to heat stress in soybean to fewer active reaction centers. Although increased ABS/RC in response to higher temperatures was expected (De Ronde et al., 2004), it is unknown whether it was due to fewer active reaction centers. In contrast, the temporal response of ABS/RC in PI-26 was unexpected as there are no studies which report temporal responses of ABS/RC. Differences in repair dynamics of PS2 in PI-26 compared to the other three genotypes may have resulted in slower, more gradual recovery of ABS/RC over the course of the 7-day treatment. Additional studies will be required to determine if the observed temporal responses are due to antenna size, active reactions centers, or other factors. Genotypes with contrasting responses such as DS25 and PI-66 should be suitable for future studies aimed at elucidating the mechanisms underpinning the different temporal responses in ABS/RC and their relevance with respect to improvement of soybean heat tolerance.

At this time, it is unclear whether the rapid return of F_V_/F_M_ and ABS/RC observed in DS25 or the slower recovery or acclimation observed for PI-26 is more advantageous for soybeans exposed to high temperatures. Information on F_V_/F_M_ dynamics over the course of several days of heat stress from other species is limited and does not provide much insight in that regard and no reports on temporal responses of ABS/RC at this time could be found. Liu and Huang (2000) reported F_V_/F_M_ from two varieties of creeping bentgrass that were exposed to control (22°C) and elevated temperature (35°C) treatments for 56 days. Temporal dynamics of F_V_/F_M_ from creeping bentgrass varieties showed steady decline to ~.5 at the end of the 56 day elevated temperature treatment while control levels remained unchanged (Liu and Huang, 2000). Differences in the temporal responses of soybean light-dependent reactions to high temperatures may be of interest as the ability to respond flexibly to perturbations in the environment was evolutionarily programmed into the photosynthesis of plants with the fast and slow component of NPQ (Krause and Weis, 1991). These components allow photoprotection to respond on different timescales (Nilkens et al., 2010), which may be critical for plant fitness as well as crop productivity (Kromdijk et al., 2016). Indeed, enhancing relaxation kinetics of NPQ by increasing expression of violaxanthin de-epoxidase, zeaxanthin epoxidase, and PS2 subunit S which increased biomass accumulation of transgenic tobacco (*Nicotiana tabacum*) (Kromdijk et al., 2016). Thus, a better understanding of the mechanisms that determine temporal dynamics of stress responses may lead to the identification of promising targets for soybean stress tolerance improvement.

### Effects of High Temperature Stress on Chloroplast Ultrastructure

Information about the impact of heat stress on chloroplast ultrastructure in soybean is largely lacking. Although Djanaguiraman et al. (2011) indicated that heat stress affected soybean chloroplast ultrastructure, including size, number, reduced grana stacking, they did not quantify the effects. Thus, to better understand the impact of heat stress on chloroplast ultrastructure as well as the relationship between ultrastructure and photosynthetic gas exchange and light reactions, chloroplast ultrastructure was assessed in DT97 and PI-66 plants exposed to the control day-time high air temperature of 28°C and the 45°C heat stress treatment for 1 day or for 7 days. The impact of heat stress was particularly pronounced on day 1 in DT97, whereas PI-66 showed little or no ultrastructure changes after 1 day of treatment (**Figure 4** and **Figure 5** and **Table 2**). In DT97, significant increases in chloroplast area (1.5×), percent grana area (1.5×), percent starch area (7.3×), total number of starch grains (3.8×), total starch area (12.1×), average area per starch grain (4.1×), total number of grana (1.4×), total grana area (1.2×), and average area per granum (1.7×) were measured. In contrast, no differences in any of these parameters, except for a 1.5-fold increase in the average area per granum, were observed in PI-66 in response to 1 day in the 45°C treatment. Given that, after 7 days of treatment, net photosynthetic rate of DT97 was more similar between the 45 and 28°C treatments than that of PI-66, the extensive chloroplast ultrastructural responses observed on day 1 in DT97 did not appear to have been detrimental to acclimation. The lack of differences between 28 and 45°C treatments on 7^th^ day in the measured grana characteristics in DT97 and the reduction in total grana area and average area per granum in PI-66, suggest a greater sensitivity of PI-66 than DT97 to several days of high temperature stress in regard to these characteristics. The increases in all grana phenotypes on the 1^st^ day of 45°C in DT97 was associated with greater F_0_, and lower F_M_ and F_V_/F_M_ values than in the 28°C treatment. Interestingly, while the grana characteristics of PI-66 were unchanged on the 1^st^ day of 45°C F_0_, F_M_ and F_V_/F_M_ values responded in the same manner as in DT97 (**Figures 3**, **5**). In contrast, starch grain number, area, and relative area per chloroplast were reduced in DT97 but not in PI-66 after 7 days of exposure to 45°C day-time elevated temperatures. These reduced levels of starch in DT97 were associated with net photosynthetic rates that did not differ between 45 and 28°C treatments, whereas net photosynthetic rates in PI-66 at 45°C were lower than at 28°C. These contrasting responses are consistent with differential sensitivity and acclimation ability of the two genotypes.

Zhang et al. (2010), reported swelling of chloroplasts in *Arabidopsis thaliana* leaves in response to 30 min of 40°C exposure, but did not report on differences in starch grain area or the effect of an extended heat stress treatment. However, their representative images had visibly more starch grain area after heat stress. The changes in starch abundance is noteworthy as starch dynamics of chloroplasts are indicative of carbon-use efficiency and can lead to limitations in growth (Ainsworth and Bush, 2011). Increases in soluble sugars can have profound effects on photosynthesis. Feedback mechanisms exist in which expression of photosynthetic genes are down regulated and starch synthesis related gene expression is increased in response to increased sugars (Sheen, 1990). Possible issues caused by heat stress may lead to decreased triose transport out of the chloroplast and/or increased sugar production which leads to metabolic feedback inhibition of photosynthesis. Given that net photosynthetic rate was maintained relative to the control treatment, the reduction of starch area in DT97 observed on the 7^th^ day of 45°C heat stress may be the result of increased sugar transport. Greater carbon export appears to be the likely reason for the reduced starch area in DT97 chloroplasts. The distinct effects of high temperature imposition on grana and starch characteristics of the two genotypes, highlight the need for further physiological and molecular characterization to elucidate the mechanisms involved in the regulation of thylakoid membrane and starch dynamics in heat stressed soybean.

## Conclusion

Exposure of soybean genotypes to elevated air temperatures for a 7-day period strongly influenced gas exchange, chlorophyll fluorescence, and chloroplast ultrastructure. Although general responses in gas exchange and chlorophyll fluorescence were similar, genotypic differences in the temporal dynamics over the course of the 7-day heat treatment were observed for numerous traits. Chloroplast ultrastructure analyses in two genotypes revealed contrasting phenotypes in chloroplast, grana, and starch characteristics in response to high temperature stress. While F_V_/F_M_ was dramatically reduced in response to high temperature stress in all genotypes on the 3^rd^ day, net photosynthetic rates were increased or not affected. The dramatic effect of 45°C on the light reactions on day 1 of the treatment was associated with increased starch and grana areas. Genotypic differences in chloroplast starch characteristics between DT97 and PI-66 were particularly pronounced on 7^th^ day of the heat treatment while net photosynthetic rates were similar between the two genotypes. The reasons underlying the contrasting responses in starch dynamics between these two genotypes are unclear but are likely due to differences in sugar transport and/or starch synthesis or turnover.

The present study provides much needed baseline information on the effects of high temperature on soybean chloroplast ultrastructure, and identified genotypes differing in the temporal characteristics of light reaction adaptation to a 7-day heat-wave simulation. Additional physiological and genetic studies are required to determine the mechanisms underlying the different responses among genotypes and the implications associated with these responses relative to heat tolerance, and to identify strategies to breed soybean for future climates.

## Data Availability Statement

The datasets generated for this study are available on request to the corresponding author.

## Author Contributions

MH planned, conducted, analyzed and provided initial manuscript draft. FF planned and provided critical review of manuscript.

## Funding

This work was partially funded by United Soybean Board project 2504.

## Conflict of Interest

The authors declare that the research was conducted in the absence of any commercial or financial relationships that could be construed as a potential conflict of interest.
